# Hysteroscopic vs. laparoscopic management of cesarian scar defect: a meta-analysis

**DOI:** 10.1186/s12884-026-09729-3

**Published:** 2026-07-28

**Authors:** Rahaf Aldiab, Majd Alswian, Ammar Halawani, Sula Almazrou, Hajar Meshal Al-Shammari, Ghaidaa Alsayed, Sarah Hussain Al-Wadai, Kayan Munahi Alotaibi, Zaid Bin Sahaq, Ralph Chalhoub, Alaa Daher, Salman Ashraf Alshahed

**Affiliations:** 1 College of Medicine, Alfaisal University, Riyadh, Saudi Arabia; 2 College of Medicine, Sulaiman Alrajhi University, Al Bukayriyah, Saudi Arabia; 3 College of Medicine, King Abdulaziz University Hospital, Jeddah, Saudi Arabia; 4 College of Medicine, Vision College, Riyadh, Saudi Arabia; 5 College of Medicine, King Khalid University, Abha, Saudi Arabia; 6 College of Medicine, King Faisal University, Hofuf, Saudi Arabia; 7 Department of Obstetrics and Gynecological Surgery, Hotel Dieu de France, Beirut, Lebanon; 8 Department of Obstetrics and Gynecological Surgery, American University of Beirut, Beirut, Lebanon; 9 Department of Obstetrics and Gynecological Surgery, King Fahad Medical City, Riyadh, Saudi Arabia

**Keywords:** Cesarian scar defect, Isthmocele, Hysteroscopy, Laparoscopy, Fertility

## Abstract

**Background:**

The prevalence of cesarian scar defects (CSD) is increasing worldwide mainly due to the increasing rates of cesarian deliveries (CD). While hysteroscopic (H) and laparoscopic (L) are both popular approaches in the management of CSD, the superiority of one over the other remains debated. Therefore, this meta-analysis will compare H and L approaches for CSD treatment.

**Methods:**

PubMed, Cochrane, and Google Scholar were queried through May 2026. Inclusion criteria consisted of studies comparing the outcomes of CSD management between H and L approaches. Extracted data included adverse events (overall complications, chronic pelvic pain, abnormal bleeding, dyspareunia, dysmenorrhea), CSD parameters (change in isthmocele depth, change in residual myometrial thickness (RMT)), surgical parameters (operative time, estimated blood loss (EBL), length of stay (LOS), and cost), and pregnancy outcomes among patients desiring pregnancy (subsequent pregnancies, abortions, premature deliveries, and term deliveries).

**Results:**

Ten studies met the inclusion criteria including 798 patients, with 361 being in the H group and 437 in the L group. Patients in the H group had a lower rate of overall complications (Odds-Ratio = 0.24, *p* = 0.02), shorter operative time (Mean difference= -55.94 min, *p*<0.001), less EBL (Mean difference= -37.73 ml; *p*<0.001), shorter LOS (Mean difference= -2.84 days; *p*<0.001), and less costs (Mean difference= $-1310; *p*<0.001). However, the H group also had less change in RMT compared to the L group (Mean difference= -2.60 mm; *p* = 0.0007). There were no differences in the remaining outcomes.

**Conclusion:**

Patients undergoing hysteroscopic management of CSD had shorter operative time, shorter length of stay, less blood loss, less costs, and less complications. However, they also had less improvement in RMT. Nevertheless, both techniques did not differ in post-operative symptoms, adverse events, or pregnancy-related outcomes, making them both a viable option for the management of CSD.

**Trial registration:**

Not applicable.

## Background

The incidence of cesarian scar defects (CSD) is on the rise due to the increasing rates of cesarean deliveries (CD) [1]. CSD ranges in the general population from 24% to 88%, depending on diagnostic method and population studied [2]. The characteristics of CSD include an empty uterus and cervical canal, the presence of a gestational sac in the anterior uterine wall with weakened myometrium between the sac and the bladder, and/or a discontinuity in the anterior wall of the uterus [3]. Several factors can contribute to its development, such as the number of prior CD, lower uterine segment incisions, and CD during advanced labor [4]. 

When symptomatic, CSD can result in a variety of gynecological symptoms, including infertility, abnormal uterine bleeding, pelvic pain, all of which can affect women’s quality of life and reproductive health [5–7]. To manage a symptomatic isthmocele, surgical interventions are often employed. Restoration of the uterine integrity is done surgically which results in alleviation of the associated symptoms [8, 9]. While a hysteroscopic approach is a minimally invasive procedure, it addresses primarily endometrial abnormalities and is often employed in smaller defects [10, 11]. On the other hand, a laparoscopic approach allows for a direct visualization and repair of deeper myometrial disruptions and is more amenable in larger or complex CSD [12, 13].

Despite the clinical importance of both techniques, the superiority of one approach over the other is still debated. While a previous meta-analysis was performed on this subject [14], they included studies comparing a hysteroscopic and laparoscopic combined approach to a hysteroscopic approach among their studies, and they did not evaluate the change in CSD parameters such as isthmocele dimensions and myometrial thickness. Therefore, our meta-analysis will perform an updated search and compare hysteroscopic and laparoscopic approaches in CSD management assessing the differences in post-operative adverse events and symptoms, operative parameters and costs, CSD parameters, and pregnancy outcomes.

## Material and methods

### Search strategy

**Fig. 1:**
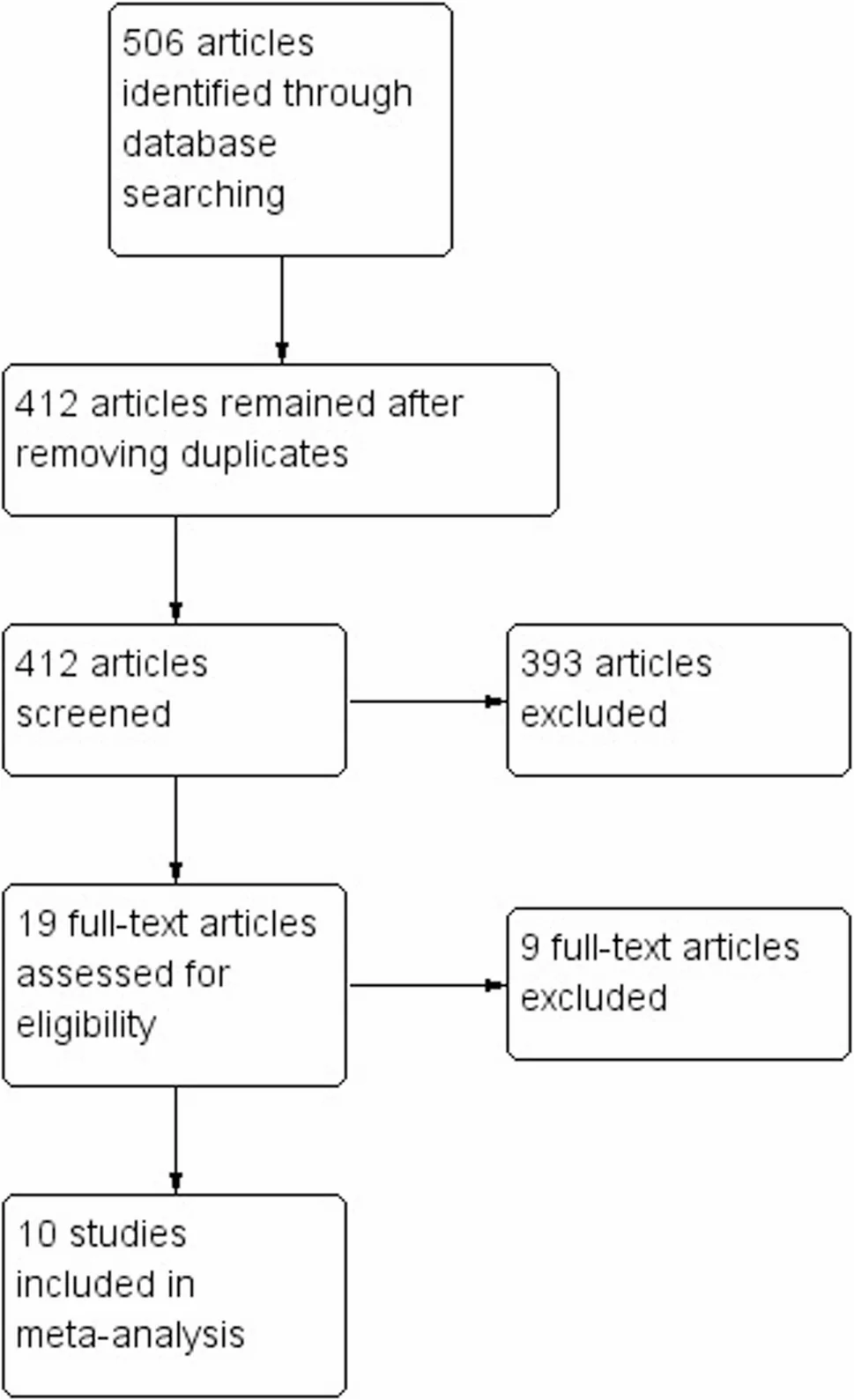
PRISMA flowchart for article selection process

Following the PRISMA guidelines, PubMed, Cochrane, and Google Scholar (pages 1–20, sorted by relevance) were accessed and explored since until May 2026 using the following keywords “Laparo*”, “hystero*”, “cesa*”, “scar”, “isthmocele”, and “niche” to find articles comparing hysteroscopic (H) and laparoscopic (L) approaches in CSD management. Additional articles were identified by reviewing reference lists from relevant articles. Two reviewers determined the eligibility of the included studies independently (Fig. 1). While one author extracted the data, another author confirmed the accuracy of the extracted data. If present, differences between the investigators were resolved by tie breaker via a third independent reviewer throughout the search and inclusion stages. This meta-analysis was not registered in PROSPERO or another registry prior to initiation.

We specifically looked at comparative articles comparing the outcomes of CSD management between H and L approaches. Inclusion criteria consisted of comparative English and non-English-language articles. There were no restrictions on language. Nevertheless, all of the included articles were in English. Therefore, specific handling for non-English articles was not required. Exclusion criteria consisted of articles combining hysteroscopic and laparoscopic approaches for the same patients, studies assessing the same cohort as other included studies, and observational single arm studies, or studies assessing outcomes after cesarean scar pregnancy surgery.

### Data extraction

The eligibility of the studies included was independently determined by two reviewers. Extracted data included adverse events (overall complications, chronic pelvic pain, abnormal bleeding, dyspareunia, dysmenorrhea), CSD parameters (change in isthmocele depth, change in residual myometrial thickness (RMT)), surgical parameters (operative time, estimated blood loss (EBL), length of stay (LOS), and cost), and pregnancy outcomes among patients desiring pregnancy (subsequent pregnancies, abortions, premature deliveries, and term deliveries).

### Risk of bias assessment

Two authors assessed the bias using the Cochrane Risk-of-Bias tool independently for included randomized controlled trials [15]. As for comparative observational studies, The ROBINS-I tool was used in a similar manner excluding articles with a critical risk of bias [16].

### Statistical analysis

Review Manager 5.4 (The Cochrane Collaboration, 2020) was implemented for statistical analysis. Mean differences (MD) with 95% CI were used for continuous data while odds ratio (OR) was utilized for dichotomous data. Heterogeneity was evaluated by Q tests and *I*^*2*^ statistics. If considerable heterogeneity was indicated by *p* < 0.05 or *I*^*2*^> 50%, random-effects model was used. Otherwise, the fixed-effect model was implemented. Statistical significance was defined as *p* < 0.05.

## Results

### Characteristics of the included studies

Database searching identified 506 articles, of which 412 remained after duplicate removal. All 412 were screened, and 393 were excluded, leaving 19 full-text articles assessed for eligibility. Of these, 9 were excluded, resulting in 10 studies that met the inclusion criteria and were included in the meta-analysis [17–26] (Fig. 1). These studies included 798 patients, with 361 being in the H group and 437 in the L group. Patients in the H group had a mean age of 35 years and a mean follow-up of 13 months, while patients in the L group had a mean age of 34 years and a mean follow-up of 16 months. The main characteristics of the included studies are summarized in Table 1. Results of the bias assessment are reported in Table 2.

**Table 1:** Characteristics of the included studies

Author	Study design	Participants		Age (years)		Follow-up			Complications	
		Hysteroscopy	Laparoscopy	Hysteroscopy	Laparoscopy	Hysteroscopy	Laparoscopy		Hysteroscopy	Laparoscopy
Ahmad et al. 2025 [17]	Randomized controlled trial	15	15	-		3 months			-	
Akay et al. 2025 [18]	Retrospective	42	84	34	33	1 year			-	
Cheng et al. 2025 [23]	Retrospective	74	80	35	34	1 year (longer for patients desiring fertility)			1 PID 1 fever 1 other	5 fevers 2 PIDs 4 others
Fatehnejad et al. 2023 [19]	Retrospective	54	45	39	38	1 year			-	
Hosseini et al. 2024 [22]	Prospective	24	22	39	38	1 year			-	
Li et al. 2014 [24]	Retrospective	24	17	-		3–16 months			-	
Li et al. 2022 [20]	Retrospective	41	48	29	30	-			-	
Zhang et al. 2021 [21]	Retrospective	35	27	32	33	18 months			-	
Q-Zhang et al. 2021 [25]	Retrospective	33	13	35	33	1–4 years			0 complications	1 complication
Zhang et al. 2016 [26]	Retrospective	19	86	-		21 months		29 months	-	

**Table 2:** Bias assessment of the included studies

Study	Confounding bias	Selection bias	Classification bias	Deviation from interventions	Missing data	Measurement of outcomes	Selective reporting	Overall
Akay et al. 2025 [18]	Moderate	Moderate	Low	Low	Moderate	Low	Low	Moderate
Fatehnejad et al. 2023 [19]	Moderate	Moderate	Low	Low	Moderate	Low	Low	Moderate
Zhang NN et al. 2021 [21]	Moderate	Low	Low	Low	Low	Low	Low	Moderate
Hosseini et al. 2024 [22]	Moderate	Low	Low	Low	Low	Low	Low	Moderate
Cheng et al. 2025 [23]	Low	Moderate	Low	Low	Moderate	Low	Low	Moderate
Li C et al. 2014 [24]	Moderate	Moderate	Low	Low	Low	Low	Low	Moderate
Zhang Q et al. 2021 [25]	Moderate	Moderate	Low	Low	Low	Low	Low	Moderate
Zhang X et al. 2016 [26]	Moderate	Moderate	Low	Low	Low	Low	Low	Moderate
Li W et al. 2022 [20]	Moderate	Moderate	Low	Low	Low	Low	Low	Moderate

### Adverse events

Two studies reported data on overall complications including 200 patients (H: 107 patients; L:93 patients), two studies reported data on pelvic pain including 156 patients (H: 57 patients; L:99 patients), five studies reported data on abnormal bleeding including 342 patients (H: 159 patients; L:183 patients), three studies reported data on dyspareunia including 255 patients (H: 111 patients; L:144 patients), and four studies reported data on dysmenorrhea including 301 patients (H:135 patients; L:166 patients).

**Fig. 2:**
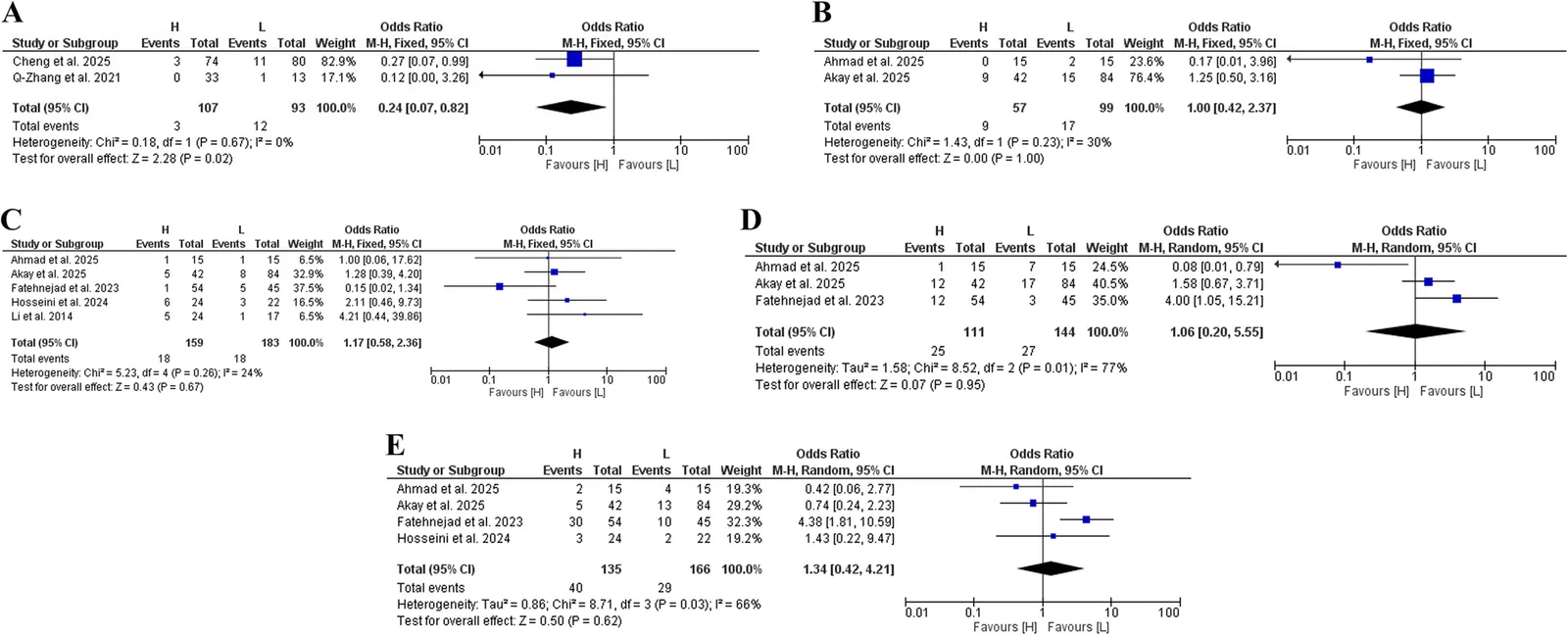
**A** Forest plot showing the difference in overall complications. **B** Forest plot showing the difference in pelvic pain. **C** Forest plot showing the difference in abnormal bleeding. **D** Forest plot showing the difference in dyspareunia. **E** Forest plot showing the difference in dysmenorrhea

Patients in the H group had a lower rate of overall complications (Odds-Ratio = 0.24; 95% CI: 0.07–0.82, *p* = 0.02, I^2^ = 0% (Fixed effect model) Fig. 2A). However, there was no difference in pelvic pain (Odds-Ratio = 1.00; 95% CI: 0.42–2.37, *p* = 1.00, I^2^ = 30% (Fixed effect model) Fig. 2B), abnormal bleeding (Odds-Ratio = 1.17; 95% CI: 0.58–2.36, *p* = 0.67, I^2^ = 24% (Fixed effect model) Fig. 2C), dyspareunia (Odds-Ratio = 1.06; 95% CI: 0.20–5.55, *p* = 0.95, I^2^ = 77% (Random effect model) Fig. 2D), or dysmenorrhea (Odds-Ratio = 1.34; 95% CI: 0.42–4.21, *p* = 0.62, (Random effect model) I^2^ = 66% Fig. 2E) between the two groups.

### CSD parameters

**Fig. 3:**
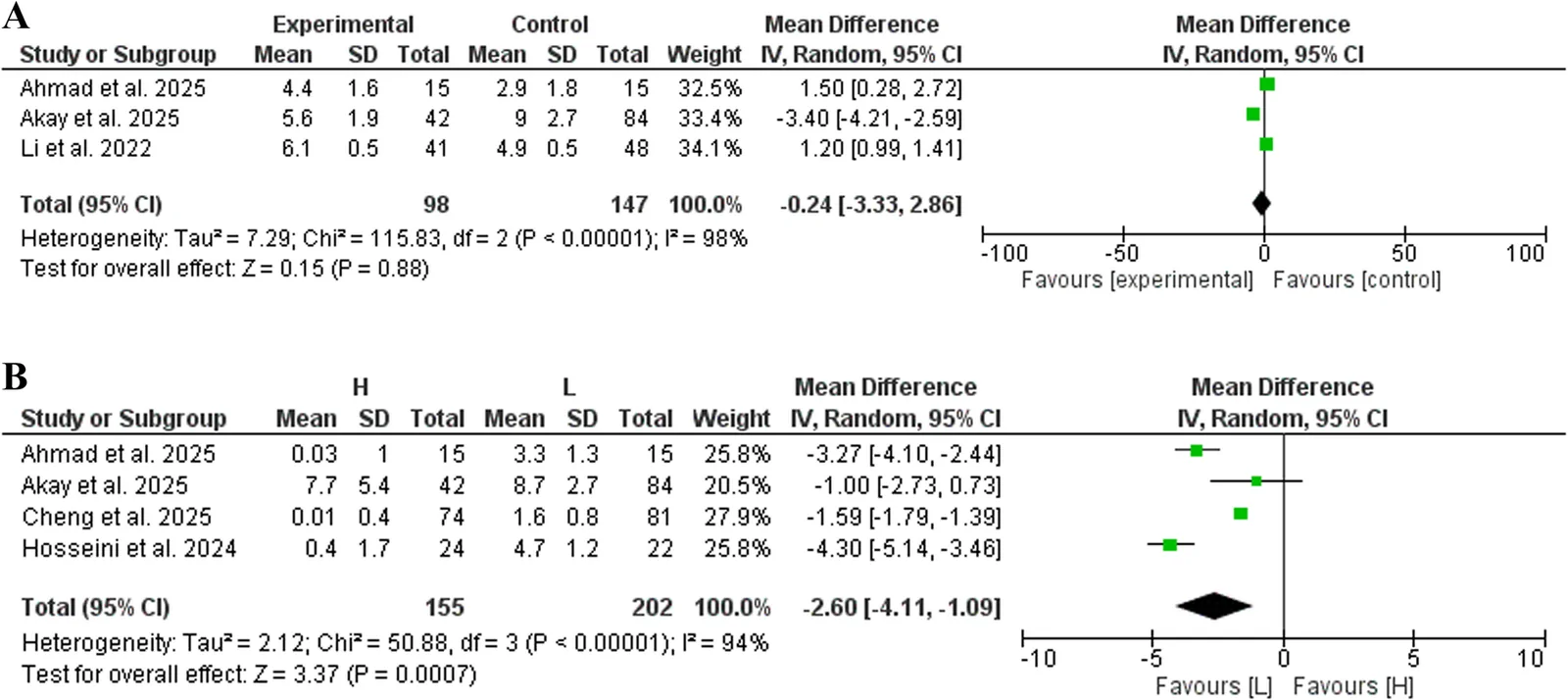
**A** Forest plot showing the difference in isthmocele depth. **B** Forest plot showing the difference in remaining myometrial thickness

Three studies reported data on change in isthmocele depth including 245 patients (H: 98 patients; L:147 patients), and four studies reported data on change in RMT including 357 patients (H:155 patients; L:202 patients). There was no difference in isthmocele depth change between the two groups (Mean difference= -0.24 mm; 95% CI: -3.33–2.86, *p* = 0.88, I^2^ = 98% (Random effect model) Fig. 3A). However, the H group had a lower change in RMT compared to the L group (Mean difference= -2.60 mm; 95% CI: -4.11– -1.09, *p* = 0.0007, I^2^ = 94% (Random effect model) Fig. 3B).

### Operative variables

**Fig. 4:**
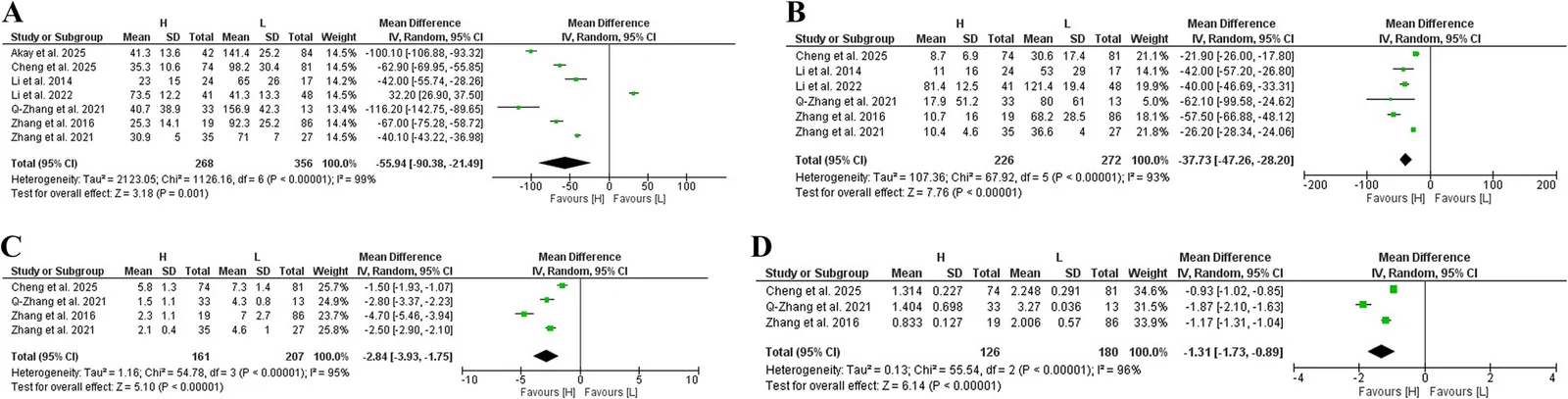
**A** Forest plot showing the difference in operative time. **B** Forest plot showing the difference in estimated blood loss. **C** Forest plot showing the difference in length of stay. **D** Forest plot showing the difference in costs

Seven studies reported data on operative time including 624 patients (H: 268 patients; L: 356 patients), six studies reported data on EBL including 498 patients (H: 226 patients; L: 272 patients), four studies reported data on LOS including 368 patients (H: 161 patients; L: 207 patients), and three studies reported data on costs including 306 patients (H: 126 patients; L: 180 patients). Compared to the L group, the H group had shorter operative time (Mean difference= -55.94 min; 95% CI: -90.38– -21.49, *p*<0.001, I^2^ = 99% (Random effect model) Fig. 4A), less EBL (Mean difference= -37.73 ml; 95% CI: -47.26– -28.20, *p*<0.001, I^2^ = 93% (Random effect model) Fig. 4B), shorter LOS (Mean difference= -2.84 days; 95% CI: -3.93– -1.75, *p*<0.001, I^2^ = 95% (Random effect model) Fig. 4C), and less costs (in $1000) (Mean difference= -1.31; 95% CI: -1.73– -0.89, *p*<0.001, I^2^ = 96% (Random effect model) Fig. 4D).

### Pregnancy outcomes

Four studies reported data on subsequent pregnancies among patients desiring pregnancies including 164 patients (H: 69 patients; L:95 patients), and two studies reported data on abortions, premature deliveries, and term deliveries including 56 pregnancies (H: 30 pregnancies; L:26 pregnancies).

**Fig. 5:**
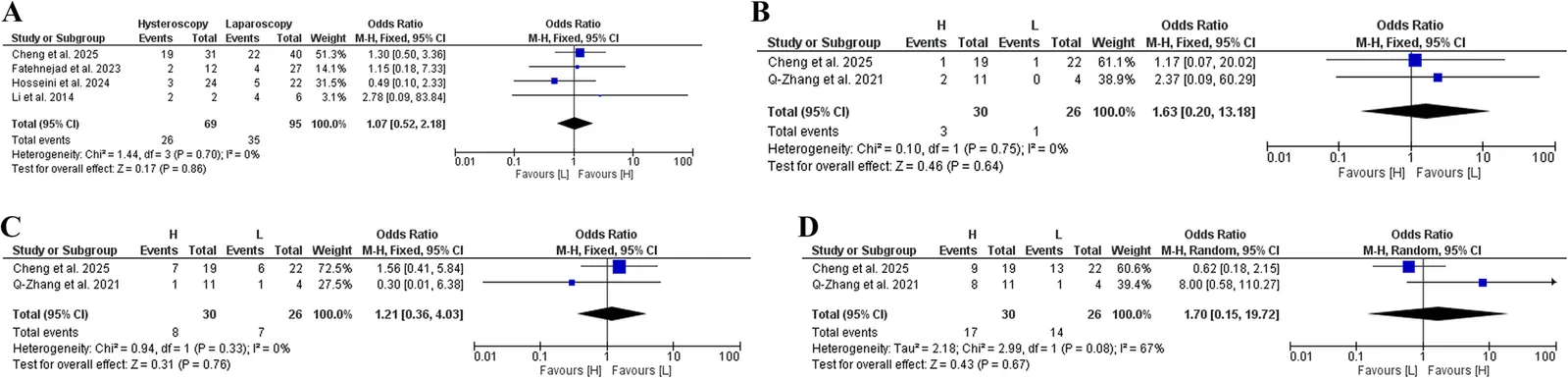
**A** Forest plot showing the difference in subsequent pregnancies. **B** Forest plot showing the difference in abortions. **C** Forest plot showing the difference in premature deliveries. **D** Forest plot showing the difference in term deliveries

There was no difference in subsequent pregnancies (Odds-Ratio = 1.07; 95% CI: 0.52–2.18, *p* = 0.86, I^2^ = 0% Fig. 5A), abortions (Odds-Ratio = 1.63; 95% CI: 0.20–13.18, *p* = 0.64, I^2^ = 0% (Fixed effect model) Fig. 5B), premature deliveries (Odds-Ratio = 1.21; 95% CI: 0.36–4.03, *p* = 0.76, I^2^ = 0% (Fixed effect model) Fig. 5C), and term deliveries (Odds-Ratio = 1.70; 95% CI: 0.15–19.72, *p* = 0.67, I^2^ = 0% (Fixed effect model) Fig. 5D) between the two groups.

## Discussion

The incidence of CSD is on the rise, ranging in the general population from 24% to 88%, depending on diagnostic method and population studied [2]. Hysteroscopic and laparoscopic approaches are both popular in the management of CSD. However, several studies reported contradictory findings regarding the superiority of one approach over the other [17–30]. In our meta-analysis, patients in the H group had a lower rate of overall complications with no difference between the two groups in symptoms, isthmocele depth, or subsequent pregnancies and their outcomes among patients desiring pregnancies. However, patients undergoing hysteroscopic management of CSD had shorter operative time, shorter length of stay, less blood loss, less costs, but also a lower improvement in RMT compared to patients undergoing laparoscopy.

Hysteroscopic approaches for the management of CSD have shown a shorter operative time, less blood loss, and shorter length of stay compared to laparoscopic approaches. A subsequent result of these differences is the lower hospital-related costs in hysteroscopic management. While Ramezani et al. reported in their meta-analysis that hysteroscopic approach had a shorter LOS and less EBL, they did not report a difference in operative time and they did not assess costs making some of our findings novel [14]. In fact, such findings further highlight hysteroscopy as a minimally invasive approach for the management of CSD as compared to laparoscopy. This reduction in operative time was around 56 min resulting in a 38 ml less blood loss, spending 2.8 days less in the hospital, and a $1310 reduction in hospital-related costs. Several studies in different specialties have shown the importance that reduced operative time has on the incidence of minor adverse events whose occurrence can result in a longer LOS, which could therefore explain the lower rate of complication in the H group [31–33]. Of note, these operative advantages should, however, be interpreted in light of the inherently less invasive and technically less demanding nature of hysteroscopy, as well as the tendency to select smaller, anatomically more favorable defects for this approach. Thus, the observed differences in operative time, EBL, LOS and cost likely reflect a combination of the procedure’s minimally invasive character and case selection rather than technical superiority of hysteroscopy.

As for symptoms resolution, we did not find differences between hysteroscopic and laparoscopic approaches. On the other hand, Ramezani et al. found a higher rate of Dysmenorrhea in patients managed with a hysteroscope [14]. This discrepancy could be explained by the fact that they only had 2 studies in their analysis with an effective of 145 patients whereas we had 4 studies in our analysis, doubling the number of patients to 300. Furthermore, contrary to Ramezani et al. showing higher pregnancy rates in laparoscopic CSD management, we did not show a difference in subsequent pregnancies or the outcomes of these pregnancies between the two approaches. An important distinction that could explain the difference in the results is that we only included patients undergoing surgery infertility, and/or desiring pregnancy post-operatively as our effective. This was not the same in the previous meta-analysis where they included the two studies, one of which by fatehnejad et al. where the authors of the meta-analysis made a mistake by reporting the “number of events” as patients who had infertility as surgery indication and not a result of the surgery [14, 19]. As for the other study they included, they put in the analysis that in the laparoscopy group, only 2 patients suffered of infertility whereas it was reported to be 5 by the study itself [14, 22]. As for RMT, although the hysteroscopic group showed a smaller change in RMT by 2.6 mm compared to the laparoscopic group, likely reflects the inherently resective nature of hysteroscopy versus the reconstructive nature of laparoscopy, rather than any true clinical inferiority. Of note, this RMT differential did not correspond to any difference in isthmocele depth change or in resolution/incidence of post-operative symptoms, and pregnancy-related outcomes.

### Potential limitations

This meta-analysis has potential limitations. As a meta-analysis, our findings depend on the variables examined by the included studies. Moreover, the results of this meta-analysis are pooled, and granular data that might affect outcomes were not accounted for. An additional limitation is the potential for clinical selection bias and baseline heterogeneity between groups, as hysteroscopic repair is generally reserved for smaller defects with adequate RMT, whereas laparoscopic repair is typically favored for larger or more complex defects with thinner RMT. Because the included studies did not consistently report baseline defect size or RMT, we were unable to perform subgroup analyses, meta-regression, or adjustment for these preoperative characteristics. Consequently, the smaller change in RMT in the hysteroscopic group may in part reflect baseline patient selection rather than the surgical technique alone. Another limitation is the heterogeneity of the included studies. However, as per the Cochrane Handbook, the heterogeneity was addressed by using a random-effects model. In addition, most of the included studies are retrospective observational studies making our meta-analysis subject to the biases that are inherent to retrospective observational studies. Lastly, pregnancy outcomes among patients desiring pregnancy were derived from only two studies comprising 56 cases. Given this small sample, these findings are likely underpowered and should be interpreted with caution, as hypothesis-generating rather than conclusive.

## Conclusion

In patients undergoing management of CSD, hysteroscopic approaches lived up to their reputation as a minimally-invasive approach showing less complications, a shorter operative time, less blood loss, shorter length of stay, and less costs. On the other hand, laparoscopic approaches results in a higher change in RMT post-operatively, an advantage inherent of the surgical technique itself. However, the latter did not translate clinically into a better resolution of post-operative symptoms, or difference in pregnancy outcomes between the two approaches. High-quality randomized controlled trials are needed in order to help surgeons guide their patients in the proper CSD management.

## Data Availability

All data generated or analysed during this study are included in this published article [and its supplementary information files].

## Abbreviations

CD: Cesarean Delivery
CI: Confidence Interval
CSD: Cesarean Scar Defect
EBL: Estimated Blood Loss
H: Hysteroscopic Approach
I²: I-squared Statistic
L: Laparoscopic Approach
LOS: Length of Stay
MD: Mean Difference
OR: Odds Ratio
PRISMA: Preferred Reporting Items for Systematic Reviews and Meta-Analyses
Q test: Cochran’s Q Test for Heterogeneity
RMT: Residual Myometrial Thickness
ROBINS-I: Risk Of Bias In Non-randomized Studies of Interventions
RCT: Randomized Controlled Trial

## References

[1] Osterman MJK. Recent Trends in Vaginal Birth After Cesarean Delivery: United States, 2016–2018. NCHS Data Brief. 2020;:1–8. 32487289

[2] Tulandi T, Cohen A. Emerging Manifestations of Cesarean Scar Defect in Reproductive-aged Women. J Minim Invasive Gynecol. 2016;23:893–902. 10.1016/j.jmig.2016.06.020. 27393285

[3] Wang D-B, Chen Y-H, Zhang Z-F, Chen P, Liu K-R, Li Y, et al. Evaluation of the transvaginal resection of low-segment cesarean scar ectopic pregnancies. Fertil Steril. 2014;101:602–6. 10.1016/j.fertnstert.2013.10.024. 24289991

[4] Bernard C, Agostini A, Bretelle F, Blanc J, Netter A. Risk factors and influence of surgical technique on the risk of caesarean scar defect formation: A systematic review of the literature. J Gynecol Obstet Hum Reprod. 2025;54:102870. 10.1016/j.jogoh.2024.102870. 39442803

[5] Murji A, Sanders AP, Monteiro I, Haiderbhai S, Matelski J, Walsh C, et al. Cesarean scar defects and abnormal uterine bleeding: a systematic review and meta-analysis. Fertil Steril. 2022;118:758–66. 10.1016/j.fertnstert.2022.06.031. 35985862

[6] Gozzi P, Hees KA, Berg C, David M, Wernecke K-D, Hellmeyer L, et al. Frequency and associated symptoms of isthmoceles in women 6 months after caesarean section: a prospective cohort study. Arch Gynecol Obstet. 2023;307:841–8. 10.1007/s00404-022-06822-8. PMC9644016 36350429

[7] Baldini GM, Lot D, Malvasi A, Di Nanni D, Laganà AS, Angelucci C, et al. Isthmocele and Infertility. J Clin Med. 2024;13. 10.3390/jcm13082192. PMC11050579 38673465

[8] Candiani M, Dolci C, Schimberni M, Bartiromo L, Villanacci R, Grisafi G, et al. Reproductive outcomes after vaginal repair of isthmocele: A preliminary study and systematic review of the literature. Eur J Obstet Gynecol Reprod Biol. 2024;296:163–9. 10.1016/j.ejogrb.2024.02.025. 38447278

[9] Etrusco A, Laganà AS, Chiantera V, Gerli S, Carugno J, Sorrentino F, et al. Efficacy, safety, and feasibility of the treatment of intrauterine pathologies with the mini-resectoscope: A systematic review. Int J Gynaecol Obstet. 2024;166:527–37. 10.1002/ijgo.15393. 38317479

[10] Al Mutairi BH, Alrumaih I. Hysteroscopy in the Treatment of Myometrial Scar Defect (Diverticulum) Following Cesarean Section Delivery: A Systematic Review and Meta-Analysis. Cureus. 2020;12:e11317. 10.7759/cureus.11317. PMC7716384 33282592

[11] Gubbini G, Centini G, Nascetti D, Marra E, Moncini I, Bruni L, et al. Surgical hysteroscopic treatment of cesarean-induced isthmocele in restoring fertility: prospective study. J Minim Invasive Gynecol. 2011;18:234–7. 10.1016/j.jmig.2010.10.011. 21354070

[12] Donnez O, Donnez J, Orellana R, Dolmans M-M. Gynecological and obstetrical outcomes after laparoscopic repair of a cesarean scar defect in a series of 38 women. Fertil Steril. 2017;107:289–e2962. 10.1016/j.fertnstert.2016.09.033. 27816234

[13] Vervoort A, Vissers J, Hehenkamp W, Brölmann H, Huirne J. The effect of laparoscopic resection of large niches in the uterine caesarean scar on symptoms, ultrasound findings and quality of life: a prospective cohort study. BJOG. 2018;125:317–25. 10.1111/1471-0528.14822. PMC5811817 28703935

[14] Ramezani Z, Goodarzi S, Rashidian P, Mohammadian S, Rastad H, Esmi M, et al. A comparison between laparoscopy and hysteroscopy approaches in the treatment of symptomatic isthmocele: A systematic review and meta-analysis. Eur J Obstet Gynecol Reprod Biol X. 2025;27:100405. 10.1016/j.eurox.2025.100405. PMC12221702 40612921

[15] Sterne JAC, Savović J, Page MJ, Elbers RG, Blencowe NS, Boutron I, et al. RoB 2: a revised tool for assessing risk of bias in randomised trials. BMJ. 2019;l4898. 10.1136/bmj.l4898. 31462531

[16] Sterne JA, Hernán MA, Reeves BC, Savović J, Berkman ND, Viswanathan M, et al. ROBINS-I: a tool for assessing risk of bias in non-randomised studies of interventions. BMJ. 2016;i4919. 10.1136/bmj.i4919. PMC5062054 27733354

[17] Ahmad RA, Refaie AN, Abdou AM, Iraki AS, Kotb MI, Elsayed RA. Hysteroscopic Resection Versus Laparoscopic Repair for Women with Caesarean Scar Defect Suffering from Postmenstrual Spotting, A Randomized Trial. Egypt J Hosp Med. 2025;99:1853–7. 10.21608/ejhm.2025.425848.

[18] Akay E, Donbaloğlu GŞ, Bayıcı N, Aslancan R. Clinical Outcomes of Isthmocele Repair Methods: A Comparison of Transvaginal, Hysteroscopic, and Laparoscopic Approaches. Anatol J Obstet Gynecol Res. 2025;1:109–17. 10.4274/anajog.galenos.2024.96168.

[19] Fatehnejad M, Hadizadeh A, Tayebi A, Ayati A, Marjani N, Gheshlaghi P, et al. Assessment of the clinical outcomes and complications of hysteroscopic and laparoscopic approaches in the treatment of symptomatic isthmocele: An observational study. Int J Gynecol Obstet. 2023;163:965–71. 10.1002/ijgo.14926. 37350282

[20] Li W, Lin T, Xie Y, Xu L, Chen Y, Zhu Y, et al. The effects of hysteroscopy and laparoscopy for scar diverticulum resection and suture on blood loss, operation time and antibiotic time. J Obstet Gynaecol (Lahore). 2022;42:3309–14. 10.1080/01443615.2022.2122708. 36200382

[21] Zhang NN, Wang GW, Yang Q. Endoscopic Treatment of Previous Cesarean Scar Defect in Women with Postmenstrual Bleeding: A Retrospective Cohort Study. J Invest Surg. 2021;34:1147–55. 10.1080/08941939.2020.1766161. 32404009

[22] Hosseini R, Parsaei M, Ali-Abad NR, Daliri S, Asgari Z, Valian Z, et al. Comparison of laparoscopic and hysteroscopic surgical treatments for isthmocele: A prospective cohort. Turk J Obstet Gynecol. 2024;21:70–7. 10.4274/tjod.galenos.2024.54006. PMC11589225 38853455

[23] Cheng S, Gao H, Li Y, Li X, Meng T, Teng D, et al. Comparison of long-term clinical effect and re-pregnant outcomes between hysteroscopic resection and laparoscopic defect repair in patients with non-severe cesarean scar defect: a retrospective study. BMC Pregnancy Childbirth. 2025;25. 10.1186/s12884-025-07667-0. PMC12076843 40369449

[24] Li C, Guo Y, Liu Y, Cheng J, Zhang W. Hysteroscopic and laparoscopic management of uterine defects on previous cesarean delivery scars. J Perinat Med. 2014;42:363–70. 10.1515/jpm-2013-0081. 24310769

[25] Zhang Q, Lei L, Zhang A, Zou L, Xu D. Comparative effectiveness of laparoscopic versus hysteroscopic approach in patients with previous cesarean scar defect: a retrospective cohort study. Ann Transl Med. 2021;9:1529–1529. 10.21037/atm-21-4339. PMC8576701 34790735

[26] Zhang X, Yang M, Wang Q, Chen J, Ding J, Hua K. Prospective evaluation of five methods used to treat cesarean scar defects. Int J Gynecol Obstet. 2016;134:336–9. 10.1016/j.ijgo.2016.04.011. 27473332

[27] Zhang X, Pang Y, Ma Y, Liu X, Cheng L, Ban Y, et al. A comparison between laparoscopy and hysteroscopy approach in treatment of cesarean scar pregnancy. Med (United States). 2020;99:E22845. 10.1097/MD.0000000000022845. PMC7581091 33120815

[28] Wang G, Liu X, Bi F, Yin L, Sa R, Wang D, et al. Evaluation of the efficacy of laparoscopic resection for the management of exogenous cesarean scar pregnancy. Fertil Steril. 2014;101:1501–7. 10.1016/j.fertnstert.2014.01.045. 24656888

[29] Sun H, Wang J, Zhou T, Liu R. Comparative Efficacy of Different Therapeutic Modalities in the Management of Cesarean Scar Pregnancy Based on Classification. J Minim Invasive Gynecol. 2025;32:970–9. 10.1016/j.jmig.2025.07.012. 40684904

[30] Yin Y, Huang L, Xu N, Ma HG, Yuan C. Comparative Analysis of the Efficacy of Four Different Surgical Modalities in the Treatment of Cesarean Scar Pregnancy: A Multicenter Retrospective Study. Adv Ther. 2025;42:1448–61. 10.1007/s12325-024-03097-0. 39853658

[31] Daniels AH, Daher M, Singh M, Balmaceno-Criss M, Lafage R, Diebo BG et al. The Case for Operative Efficiency in Adult Spinal Deformity Surgery. Spine (Phila Pa 1976). 2023. 10.1097/BRS.0000000000004873. 37942794

[32] Catanzarite T, Saha S, Pilecki MA, Kim JYS, Milad MP. Longer Operative Time During Benign Laparoscopic and Robotic Hysterectomy Is Associated With Increased 30-Day Perioperative Complications. J Minim Invasive Gynecol. 2015;22:1049–58. 10.1016/j.jmig.2015.05.022. 26070725

[33] Rottenstreich M, Sela HY, Shen O, Michaelson-Cohen R, Samueloff A, Reichman O. Prolonged operative time of repeat cesarean is a risk marker for post-operative maternal complications. BMC Pregnancy Childbirth. 2018;18:477. 10.1186/s12884-018-2111-8. PMC6280485 30514224

